# Study of surface carbohydrates in *Galba truncatula* tissues before and after infection with *Fasciola hepatica*

**DOI:** 10.1590/0074-02760160141

**Published:** 2016-07-04

**Authors:** Katya Georgieva, Liliya Georgieva, Yana Mizinska-Boevska, Stoyanka R Stoitsova

**Affiliations:** 1 Bulgarian Academy of Sciences, Institute of Biodiversity and Ecosystem Research, Department of Animal Diversity and Resources, Sofia, Bulgaria; 2 Bulgarian Academy of Sciences, Institute of Experimental Morphology, Pathology and Anthropology with Museum, Sofia, Bulgaria; 3 Bulgarian Academy of Sciences, Institute of Microbiology, Sofia, Bulgaria

**Keywords:** Galba truncatula, Fasciola hepatica, surface carbohydrates, lectin labelling, carbohydrate similarity

## Abstract

The presence and distribution of surface carbohydrates in the tissues of *Galba truncatula* snails uninfected or after infection with *Fasciola hepatica* as well as on the surface of the snail-pathogenic larval stages of the parasite were studied by lectin labelling assay. This is an attempt to find similarities that indicate possible mimicry, utilised by the parasite as an evasion strategy in this snail-trematode system. Different binding patterns were identified on head-foot-mantle, hepatopancreas, genital glands, renopericardial complex of the host as well as of the snail-pathogenic larval stages of *F. hepatica*. The infection with *F. hepatica* leads to changes of labelling with *Glycine max* in the head-mantle cells and *Arachis hypogaea* in the tubular epithelium of the hepatopancreas. The lectin binding on the other snail tissues is not changed by the development of the larvae. Our data clearly demonstrated the similarity in labelling of *G. truncatula* tissues and the surface of the snail-pathogenic larval stages of *F. hepatica*. The role of glycosylation of the contact surfaces of both organisms in relation to the host-parasite interactions is also discussed.

Several freshwater snails act as obligatory intermediate host in the complex life cycle of the liver fluke, *Fasciola hepatica*, a helminth parasite affecting wild and domestic animals and humans worldwide (Mas-Coma et al. 2009). Different genera of lymnaeid snails including *Galba* (Mas-Coma et al. 2009), *Omphiscola* (Dreyfuss et al. 2003), *Stagnicola* (Relf et al. 2009), *Pseudosuccinea* (Vázquez et al. 2014), *Radix* (Caron et al. 2014), etc., can be intermediate hosts where asexual reproduction of the parasite takes place. *Galba truncatula* is the principle intermediate host within Europe (Bargues et al. 2001). The free living parasite larvae (miracidia) penetrate the snail head-foot-mantle surface. During penetration miracidia lose their ciliated coat and newly formed sporocysts enter the invertebrate host. Proliferative asexual development of sporocysts leads to the next stage, the rediae. The rediae can produce up to fourth daughter generations, which in turn produce cercariae (Rondelaud et al. 2009). The cercariae perform a complex migration in snail tissues before being shed in the environment, where they transform to metacercariae, the invasive larvae for the definitive hosts. Sporocysts, rediae and cercariae are located between or in visceral organs of the snail. They are in abundance in zones surrounding the hepatopancreas (digestive gland), genital (albumen, nidamental and prostate glands) and renopericardial complexes.

The search for new opportunities for control of this major trematode infection draws the attention to the specific mechanisms enabling the parasite to survive and multiply inside the invertebrate host.

Mollusks have an internal defense system which is able to recognise and respond to invading parasites (Van der Knaap & Loker 1990, Bayne 2009). Immune recognition is considered to be carried out by pattern recognition receptors (PRRs) (Janeway & Medzhitov 2002), which bind to structures referred to as pathogen-associated molecular patterns (PAMPs) (Janeway 1989). In the context of the snail-trematode interactions, currently known PRRs with larval trematode-binding capabilities include the large class of carbohydrate binding proteins, or lectins (Yoshino & Coustau 2011 , Adema & Loker 2015). The ligand molecules of these lectins are the carbohydrate residues of glycoconjugates situated at the larval surface or released in the host environment. Numerous studies have demonstrated the participation of larval surface carbohydrates in immune recognition and the activation of the signaling pathways involved in the immune response, but also in the mechanisms that allow the parasites to evade snail defense (Yoshino & Coustau 2011). The general hypotheses of parasite-host immune interactions are based on lectin-carbohydrate interactions, namely molecular mimicry (Bayne 2009), compatibility polymorphism (Roger et al. 2008) or modulation of snail immune cells‘ reactivity (Yoshino & Coustau 2011).

A special role of surface carbohydrates of helminth parasites in the mechanisms allowing modulation of the immunе response of the snail host is studied more detailed in *Biomphalaria-Schistosoma* system (Coles et al. 1988, Uchikawa & Loker 1991, Nyame et al. 2002 , Lehr et al. 2007, 20, 2008, Peterson et al. 2009 , Yoshino et al. 2013), as well in other snail-trematode associations (Iakovleva & Gorbushin 2005 , Kawasaki et al. 2013). Despite of the broad prevalence of *F. hepatica* in the world, to this time there are no data of the immunological interactions between larval stages of the parasite and its intermediate snail host. In the present study we compare the lectin-binding characteristics of *G. truncatula* tissues, before and after infection with *F. hepatica* and identify the carbohydrate residues on the surface of the snail-pathogenic larval stages of *F. hepatica*, namely sporocysts, rediae and cercariae. This approach allows the detection of common surface saccharides of the snail tissues and the parasite larvae developed in the invertebrate host as well as the changes of the surface glycosylation of the host tissues in the course of the infection with *F. hepatica*. This is an attempt to indicate carbohydrate mimicry, utilised by the parasite as an evasion strategy in *G. truncatula - F. hepatica* system.

## MATERIALS AND METHODS

*Snails and parasites* - *G. truncatula* were cultivated in our laboratory. *F. hepatica* were obtained from experimental life cycle of the parasite maintained using *Galba* snails as intermediate and male *Wistar* rats as definitive hosts. The procedures have been previously described in detail by Georgieva et al. (2012). Tissue samples of adult *G. truncatula* snails from either uninfected snails, or snails infected with *F. hepatica* were taken eight, 14 and 50 days post infection. The intervals correspond to the time when the sporocysts, rediae, or cercariae were isolated. Larval forms of the parasite were collected after careful detachment of the shell from the snail body. Sporocysts are located in the hepatopancreas and were separated from this tissue. Larger in size rediae and cercariae are in abundance around the hepatopancreatic tubules and a less in spaces between other organs and were easy collected after removing of the shell. Three snails were used for each tissue staining procedure and approximately thirty larvae, obtained from five (for rediae and cercariae) or more (for sporocysts) infected snails were used for each labelling procedure.

*Lectin labelling of tissue sections and whole mount larvae* - The uninfected and infected snails were fixed in 4% paraformaldehyde in phosphate-buffered saline (PBS) (0.1 M Na_2_HPO_4_, 0.1 M NaH_2_PO_4_.2H_2_O, 0.15 M NaCl, pH 7.4) for 3 h at 4ºC. After careful removal of the shells, the fixation was continued for 1 h. Then, the snails were well washed with PBS, dehydrated and embedded in paraffin. Serial sections (6 µM thick) were collected on microscope slides without additives and allowed to dry overnight. The sections were dewaxed and hydrated. After blocking of non-specific binding with 5% bovine serum albumin (Sigma-Aldrich) the samples were labelled with lectin-fluorescein isothiocyanate (FITC) conjugates following the procedures described by Georgieva et al. (2012). The applied lectins (Sigma-Aldrich or Vector Labs), their major carbohydrate specificities and the final concentrations in PBS [or PBS supplemented with 0.1 mM CaCl_2_ and MnCl_2_ for Concanavalin A (ConA)] are listed in Table I. Іncubations took place in the dark, for 1 h, at room temperature. The treated sections were washed with PBS and observed using a Leica DM 5000B fluorescence microscope. Two control tests were applied: (i) inhibitory controls: preincubation of the lectins with inhibitory sugars (see Table I); (ii) estimation of autofluorescence: omission of the lectin-FITC conjugate during incubation.

**TABLE I:** Lectins used in this study, their carbohydrate-binding specificities, the concentration used, the corresponding inhibitory sugars and their concentration

Lectin	Specificity	Lectin concentration (μg/mL)	Inhibitory sugar	Sugar concentration used in inhibitory tests
ConA	α-Man, α-Glc	20	MetMan	0.2 M
LCA	α-Man	20	MetMan	0.2 M
WGA	(GlcNAc)_2_	20	GlcNAc	0.5 M
LEL	(GlcNAc)_3_	20	GlcNAc	0.2 M
SBA	GalNAc	20	GalNAc	0.2 M
HPA	GalNAc	20	GalNAc	0.2 M
PNA	β-Gal(1→3)GalNAc	20	Gal	0.2 M
UEA-I	α-L-Fuc	20	Fuc	0.2 M

Lectins: ConA - Concanavalin A; HPA - *Helix pomatia*; LCA - *Lens culinaris;* LEL - *Lycopersicon esculentum*; PNA *- Arachis hypogaea*; SBA - *Glycine max*; UEA-I - *Ulex europaeus*; WGA - *Triticum vulgaris*; Sugars: Fuc - α-L-fucose; Gal - galactose; GalNAc - N-acetyl-D-galactosamine; Glc - α-glucose; GlcNAc - N-acetyl-D-glucosamine; Man - α-mannose; MetMan - methyl α-D-mannopyranoside.

Sporocysts, rediae and cercariae were collected from carefully broken infected snails in saline (5 mM Hepes, 36 mM NaCl, 2 mM KCl, 2 mM MgCl_2_, 4 mM CaCl_2_, pH 7.8) and fixed in 4% paraformaldehyde and 0.1% glutaraldehyde in PBS for 2 h, at 4ºC. After a buffer rinse, the fixed parasites were incubated with lectin-FITC conjugates as above, transferred onto microscopic slides, covered with cover slips and observed with a fluorescence microscope.

## RESULTS

**Fig. 1:**
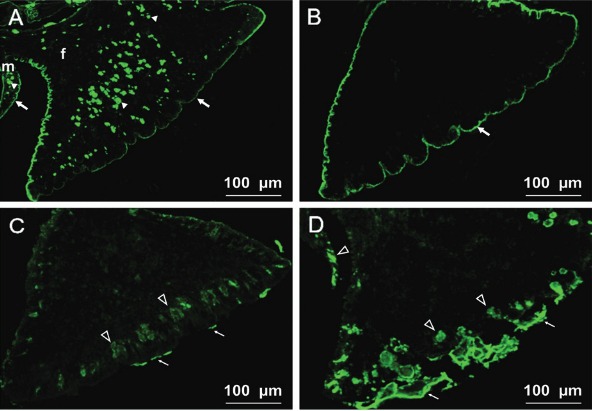
: lectin-fluorescein isothiocyanate labelling of the foot-mantle tissues of uninfected (A) and eight days post infected (B, C, D) snails *Galba truncatula* with larvae of *Fasciola hepatica*. (A) *Lycopersicon esculentum* positive reaction on the cells (arrowheads) in foot (f) and mantle (m) and also on the surface epithelia (arrows); (B) presence of *Helix pomatia* binding sites on the foot epithelium (arrow); (C) positive *Arachis hypogaea* labelling on the foot subepithelial gland cells (transparent arrowheads) and mucus (thin arrows); (D) an intense labelling with *Ulex europaeus*-I of the subepithelial gland cells in the foot and mantle (transparent arrowheads) and the mucus of the foot end (thin arrows).

*Lectin labelling of tissue sections of uninfected and infected snails* - *Staining of head-foot-mantle tissues* - Each lectin displayed a specific pattern of staining on the head-foot-mantle tissues. The binding sites were observed on the different types of gland cells, on the epithelia and the mucus of the snail foot. The mannose/glucose specific lectins ConA and *Lens culinaris* (LCA) did not label the head-foot-mantle region of uninfected and infected snails (Table II). A clear staining with the N-acetylglucosamine-specific *Triticum vulgaris* (WGA) and *Lycopersicon esculentum* (LEL) (Fig. 1A) was found on the cells in the head-foot-mantle tissues as well as on the epithelia. Both lectins show similarity in their reactions in uninfected and infected snails (see Table II). Staining with the N-acetylgalactosaminе-specific lectins *Glycine max* (SBA) and *Helix pomati*a (HPA) and the galactose-specific *Arachis hypogaea* (PNA) exhibited specific patterns. Sites for SBA were present only in the head and mantle cells of uninfected and up to 14 days post-infected snails (Table II). An intense HPA (Fig. 1B) binding was observed on the head-foot-mantle epithelial surfaces. Binding sites for PNA were detected on the subepithelial gland cells and mucus of the foot end (Fig. 1C).

**TABLE II:** Lectin labelling of tissues of uninfected snails *Galba truncatula* and infected with *Fasciola hepatica*

Lectins	Head -Foot -Mantle												Hepatopancreas								Hermaphroditic gland (Gonad, ovotestis)				Genital glands				Renopericardial complex			

	Gland cells				Epithelia				Mucus				Tubular epithelium				Tubular wall															

	Uninfected	8 days pi	14 days pi	50 days pi	Uninfected	8 days pi	14 days pi	50 days pi	Uninfected	8 days pi	14 days pi	50 days pi	Uninfected	8 days pi	14 days pi	50 days pi	Uninfected	8 days pi	14 days pi	50 days pi	Uninfected	8 days pi	14 days pi	50 days pi	Uninfected	8 days pi	14 days pi	50 days pi	Uninfected	8 days pi	14 days pi	50 days pi
ConA	-	-	-	-	-	-	-	-	-	-	-	-	-	-	-	-	-	-	-	-	-	-	-	0	-	-	-	-	-	-	-	-
LCA	-	-	-	-	-	-	-	-	-	-	-	-	++	++	++	++	++	++	++	++	+	+	+	0	+	+	+	+	+	+	+	+
WGA	^1^++	^1^++	^1^++	^1^++	++	++	++	++	-	-	-	-	+	+	+	+	-	-	-	-	+	+	+	0	++	++	++	++	++	++	++	++
LEL	^1^++	^1^++	^1^++	^1^++	++	++	++	++	-	-	-	-	++	++	++	++	++	++	++	++	+	+	+	0	++	++	++	++	++	++	++	++
SBA	^2^+	^2^+	^2^+	-	-	-	-	-	-	-	-	-	-	-	-	-	-	-	-	-	-	-	-	0	-	-	-	-	-	-	-	-
HPA	-	-	-	-	++	++	++	++	-	-	-	-	-	-	-	-	-	-	-	-	+	+	+	0	-	-	-	-	-	-	-	-
PNA	^3^+	^3^+	^3^+	^3^+	-	-	-	-	+	++	+	+	-	±	+	+	-	-	-	-	-	-	-	0	^4^+	^4^+	^4^+	^4^+	+	+	+	+
UEA-I	^3^++	^3^++	^3^++	^3^++	-	-	-	-	++	++	++	++	-	-	-	-	++	++	++	++	+	+	+	0	++	++	++	++	+	+	+	+

Intensity of staining: ++ = intense; + = moderate; ± = weak; - = none; 0 - not tested; pi - post infection; ^1^- the label is present on the gland cells in the foot and mantle; ^2^- the label is on the gland cells in the head and mantle; ^3^-the label is on the subepithelial gland cells in the foot; ^4^-labelling of the albumen gland.

A strong labelling with the fucose-specific *Ulex europaeus* (UEA-I) was observed on the subepithelial gland cells in the foot and mantle and on the mucus of the foot end (Fig. 1D). No differences in labelling of uninfected and infected snails were observed. The specificity of the binding reactions was confirmed by the negative results of control tests.

**Fig. 2:**
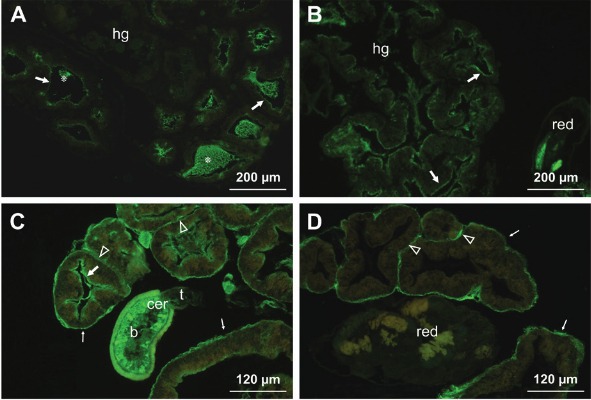
: lectin-fluorescein isothiocyanate labelling of the hepatopancreas and hermaphroditic gland tissue of infected *Galba truncatula* snails with *Fasciola hepatica*. (A) *Arachis hypogaea* (PNA) labelling of the eight days post infected snails. The epithelium lining the hepatopancreatic tubules (arrows) appeared weakly stained but the content of the lumen (*) show reactivity to the lectin; hg - the hermaphroditic gland embedded in the hepatopancreas; (B) an increased PNA labelling of the tubular epithelium (arrows) in 50 days post infected snails; the hermaphroditic gland (hg) is atrophied, no reaction on the surface of the redia (red) is observed; (C) an intensive labelling of *Lens culinaris* on the tubular epithelium (arrow), wall (thin arrows), an intertubular connective tissue (transparent arrowheads) and the cercarial body (b), located between tubules, 50 days post infection; cer - cercaria, t - cercarial tail; (D) *Ulex europaeus*-I staining on the tubular walls (thin arrows) and the intertubular connective tissue (transparent arrowheads) and no reaction on the redia (red), 50 days post infection.

*Staining of hepatopancreas and hermaphroditic gland (gonad, ovotestis) tissues* - Incubation in the hepatopancreas sections with the applied lectins resulted in positive reactions with LCA, WGA, LEL, PNA and UEA-I. We observed a difference in labelling with PNA of uninfected, early infected and other groups of infected snails. In uninfected snails, PNA staining was observed on content filling the lumen of the hepatopancreatic tubules. In eight days post infected snails, a slight labelling of the epithelium lining the tubules (tubular epithelium) was observed (Fig. 2A). The progress of infection resulted in the enrichment of sites for this lectin (Fig. 2B). The labelling patterns of the other tested lectins did not change with parasite infection (Table II). Incubation with LCA (Fig. 2C) and LEL led to intensive labelling of the tubular epithelium and wall as well as the intertubular connective tissue (loose connective tissue between the tubules). WGA labelling was marked in the tubular epithelium only (Table II). A clear staining with UEA-I was observed on the tubule walls and intertubular connective tissue (Fig. 2D).

In our experiment, analysis of sections of uninfected and infected snails showed that the hermaphroditic gland, embedded in the hepatopancteas, undergoes strong atrophic changes due to the presence of larval forms of *F. hepatica*. In uninfected and up to 14 days post infected snails, the gland structure is preserved and the sites for LCA, WGA, LEL, HPA and UEA-I were found. After 50 days of infection, the gland tissue is destructed (see Fig. 2B).

Specificity of the labelling reactions was confirmed by the blocking procedures with specific sugars and absence of autofluorescence.

**Fig. 3:**
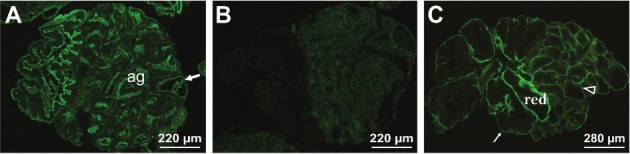
: lectin-fluorescein isothiocyanate labelling of the genital gland tissues in 50 days infected snails *Galba truncatula* with larvae of *Fasciola hepatica*. (A) A positive *Lycopersicon esculentum* staining on the epithelium, lined the gland tubules (arrow); ag - albumen gland; (B) presence of *Arachis hypogaea* binding sites in the cells of the albumen gland; (C) an intensive labelling with *Ulex europaeus*-I of the tubular walls (thin arrow) and intertubular connective tissue (transparent arrowhead). The (red) is inserted into tubule redia, which is not stained.

*Staining of genital (albumen, nidamental and prostate) gland tissues* - The binding sites for LCA, WGA, LEL, PNA and UEA-I were observed on the genital glands sections and there was no marked difference of labelling between the uninfected and infected snails. Reactions with LCA and LEL (Fig. 3A) were detected on the tubular epithelia, the content of the lumen and the connective tissue between tubules. The labelling with WGA was different, the albumen gland was not stained, but staining was detected on the epithelium of the prostate gland tubules. However, the albumen gland cells were labelled with PNA and no staining of the other genital tissues was observed (Fig. 3B). The walls and intertubular connective tissue of the genital gland tubules were intensively labelled with UEA-I (Fig. 3C).

**Fig. 4:**
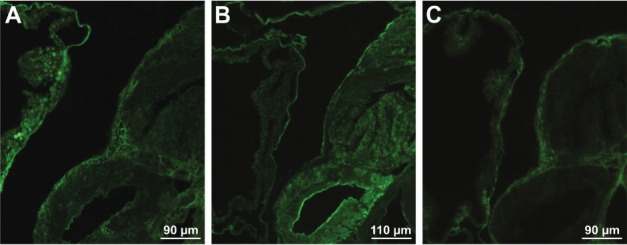
: lectin-fluorescein isothiocyanate labelling of the renopericardial tissues of *Galba truncatula* snails, on day 50 post infection with *Fasciola hepatica*. (A) Moderate staining with *Lens culinaris*; (B) labelling with *Triticum vulgaris*; (C) positive reaction with *Ulex europaeus-*I on the wall of tissues.

*Staining of renopericardial complex* - These visceral tissues were positively labelled by LCA (Fig. 4A), WGA (Fig. 4B), LEL, PNA and UEA-I (Fig. 4C), and no marked differences were found in the staining of uninfected and infected snails. Lectins were bound to different parts of tissues. The reactions with WGA and PNA were intensive, binding of UEA-I was mainly limited on the tissue walls. Control reactions were negative, confirming the specificity of the lectin labelling.

The lectin labelling results obtained for uninfected and infected snails are summarised in Table II.

*Lectin labelling of F. hepatica sporocysts, rediae and cercariae* - Each larval stage of *F. hepatica*, developed in *G. truncatula* snails, exhibited specificity in labelling with the applied lectins.

**Fig. 5:**
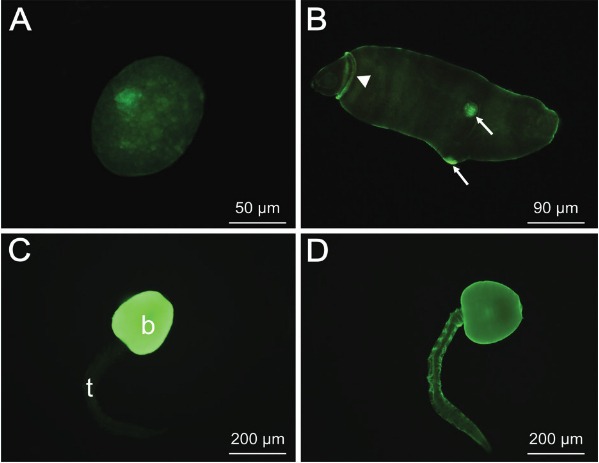
: labelling of an entire sporocyst (A), redia (B) and cercariae (C-D) of *Fasciola hepatica* with fluorescein isothiocyanate-conjugated lectins. (A) The reaction on the sporocyst surface with *Arachis hypogaea* (PNA); (B) labelling of redia with *Lens culinaris*, more intense on the head collar-like structure protruding between the head and body (arrowhead) and the lateral body projections (arrows); (C) labelling of the cercarial body surface (b) with *Glycine max* and no reaction on the cercarial tail (t); (D) localisation of the binding sites for PNA on the cercarial body and tail.

Sporocysts bound six out of eight tested lectins. On the surface of the entire larvae and tissue sections were found carbohydrate residues recognised by ConA, LCA, WGA, LEL, HPA and PNA (Fig. 5A). No labelling occurred with SBA and UEA-I. The results for lectin binding of the whole sporocysts and tissue sections showed no significant differences.

The surface of the rediae of *F. hepatica* was labelled with four out of the eight tested lectins. Positive results for LCA, WGA, LEL and HPA were observed. Incubations with LCA (Fig. 5B), WGA and LEL resulted in labelling of the entire larvae. Even more intense labelling was seen on parts of the redial body such as the collar-like structure protruding around the redial head and the lateral projections. The reaction with HPA resulted in uniform staining of the whole redial surface. No sites for the other tested lectins, ConA, SBA, PNA and UEA-I were found.

The surface of the cercariae of *F. hepatica* was recognised by a different set of lectins, in comparison with the sporocysts and rediae (Table III). Furthermore, the binding pattern of some lectins differed between the cercarial body and tail. On the body were displayed binding sites for ConA, LCA (see Fig. 2C), WGA, SBA (Fig. 5C) and PNA (Fig. 5D). The surface of the entire cercariae (body and tail) was labelled only by WGA and PNA.

**TABLE III:** Lectin binding results in *Fasciola hepatica* larvae

Lectin	Miracidia*	Sporosysts	Rediae		Cercariae	
	
			Body	Head collar and projections	Body	Tail
ConA LCA	± ±	± +	- ±	- ^1^++/ ^2^+	+ ^1^+/ ^2^++	- -
WGA LEL	++ ++	++ ++	+ +	^1^++/ ^2^+ ^1^++/ ^2^+	++ -	++ -
SBA HPA PNA	- - +	- + +	- ++ -	- ++ -	^1^++/ ^2^+ - ++	- - ++
UEA-I	-	-	-	-	-	-

Intensity of staining: ++ = intense; + = moderate; ± = weak; − = none. * Georgieva et al. (2012, 2014); ^1^it refers to the labelling of entire larvae; ^2^the labelling of the tissue sections.

Specificity of the all labelling reactions was confirmed by the inhibitory control with specific sugars and the absence of autofluorescence.

Lectin labelling of the *F. hepatica* larvae are summarised in Table III.

## DISCUSSION

The effect of the larval forms of *F. hepatica* on the morphology and the metabolic processes of the host tissues was described in detail in several previous studies (Humiczewska & Taracha 1985, 1987, Lapeta 2001, 20, 2003, Moore & Halton 1973). Our study is focused on the presence of surface carbohydrates in the tissues of uninfected and infected *G. truncatula* snails as well as the glycosylation pattern of snail-pathogenic larval stages of the *F. hepatica*.

The head-foot-mantle is the site where the invasive larvae of the *F. hepatica,* the miracidia, penetrate the snail. The results reveal the occurrence of the various carbohydrate residues present as well as in the different types of gland cells, the surface epithelia and in the mucus of the foot end. The gland cells, located within head-foot-mantle comprise of segments which are motile. During moving to the surface, their product (mucus) undergoes changes before being discharged on the body surface (Portela et al. 2012). Our data showed that these cells in *G. truncatula* are labelled with N-acetylglucosamine specific WGA and LEL, but no labelling of the secreted mucus with these lectins is observed.

Another type of gland cell is located under the epithelial surface of head-foot-mantle and our results showed that they contained glycoconjugates recognised by galactose - and fucose - specific PNA and UEA-I. An abundance of sites for UEA-I and to a lesser extent, PNA are also present in the secreted mucus. It is generally accepted that glycoconjugates in the mucus and on the epithelial surface play a role in the attraction and attachment of miracidia to the host (Haas 2003). Our results are in accordance with those of Kalbe et al. (2000) which identified fucose and galactose as components of the “miracidia-attracting glycoproteins”, isolated from snail-conditioned water of uninfected *G. truncatula*.

In this work, labelling with ConA and LCA revealed the absence or minimal amount of glycoconjugates containing mannose/glucose residues in the head-foot-mantle tissues of uninfected and infected *G. truncatula*.

The infection with *F. hepatica* did not change the glycosylation of the epithelial surfaces as well as the secreted mucus of the head-foot-mantle region of *G. truncatula*. Furthermore, the labelling of the gland cells with WGA, LEL, PNA and UEA-I do not differ between uninfected and infected snails. However, a different result was obtained for SBA. This lectin was found to bind to cells located in the head and mantle of uninfected and up to 14 days post-infected snails. Obviously, prolonged infection with *F. hepatica* leads to disappearance of sites for SBA. This probably reflects changes in the endocrine regulation of some metabolic processes of the snail, as consequence a parasite invasion.

In studies on the infectivity of the *F. hepatica* miracidia, Christensen et al. (1976) found that the attachment and penetration of *G. truncatula* tissues are not influenced by an already existing infection with the same parasite, regardless of its developmental stage. The current data show that the parasite infection does not change the glycosylation of the contact surfaces as well as the secreted mucus, which implies a role of carbohydrates in providing the miracidia with the correct conditions for attachment to the snail host.

The hepatopancreas of *G. truncatula* and the close area of the genital glands are referred locations for larvae of *F. hepatica*, especially for sporocysts, although parasites were found through all the viscera. Lectin labelling of the hepatopancreas tissue revealed the occurrence of mannose/glucose, N-acetylglucosamine and fucose residues, recognised by LCA, WGA, LEL and UEA-I. The binding pattern of these lectins does not differ in the uninfected and infected hepatopancreas. However, the progress of infection with *F. hepatica* leads to the appearance of galactose residues, bound by PNA. Our data showed that uninfected hepatopancreas tissue did not react with this lectin, but after infection, when the parasite larval forms were developed, the epithelium of the digestive tubules displayed sites for this lectin.

The hermaphroditic gland, embedded in the hepatopancreas, is the tissue most affected by parasite invasion. In uninfected and early infected snails, the hermaphroditic gland is labelled with LCA, WGA, LEL, HPA and UEA-I. The multiplication of the parasite larvae, however, leads to massive destruction of the gland tissue and staining with aforementioned lectins was not observed.

The genital (albumen, nidamental and prostate) gland complex maintained its structural integrity during invasion with larvae of *F. hepatica* and was labelled with a different set of lectins. The albumen gland reacted with LCA, LEL, PNA and UEA-I, revealing the presence of mannose/glucose, N-acetylglucosamine, galactose and fucose residues. WGA binding was observed in the lumen of the prostate gland. Development of larval forms of *F. hepatica* did not lead to a change of the lectin labelling in the genital gland tissues.

The renopericardial complex of uninfected and infected *G. truncatula* was labelled by LCA, WGA, LEL, PNA and UEA-I, indicating the presence of mannose/glucose, N-acetylglucosamine, galactose and fucose residues. In the proximal part of the kidney, the labelling with LCA and UEA-I was mainly on the wall. Our results show that the infection with *F. hepatica* does not affect the lectin binding of this visceral complex.

Lectin labelling of the entire larvae and tissue sections of *F. hepatica*, developed within the common snail host *G. truncatula*, revealed specific surface glycosylation of each of the stages. The sporocysts of *F. hepatica* are formed after transformation of the miracidia during penetration of the snail body. Here, on the sporocyst`s surface we identified mannose/glucose, N-acetylglucosamine, N-acetylgalactosamine and galactose residues. Previously, we studied the surface glycosylation of the miracidia using the same set of lectins (Georgieva et al. 2012, 2014), and found mannose/glucose, N-acetylglucosamine and galactose residues. It is obvious, that transformation to the sporocyst stage is followed by an expansion in carbohydrate diversity as seen by the appearance of N-acetylgalactosamine residues recognised by HPA. The differences in surface glycosylation of miracidia and sporocysts probably reflect the different functions of the parasite surface saccharides in the interactions with the snail host. Free-living miracidia penetrate the snail via the epithelium of the head-foot-mantle. The carbohydrates on the surface of invading pathogens are thought to bind to specific host molecules and affect the outcome of the infection (El-Ansary 2003). It is possible that interactions between miracidial carbohydrates and snail carbohydrate-binding molecules lead to the initiation of the transformation into sporocysts, as indicated in our *in vitro* study (Georgieva et al. 2012). Sporocysts live in snail tissues and are subjected to the snail internal defense system. Thе snail recognition system includes carbohydrate-binding molecules, or lectins, which can bind/recognise larval carbohydrate structures and initiate anti-parasite responses (Yoshino et al. 2001, Bayne 2009). In a lectin-carbohydrate recognition system, the presence of identical carbohydrate residues on the larval parasites and surrounding host tissues or cells (carbohydrate mimicry) protects the larvae from immune recognition (Lehr et al. 2007, 2008, Kawasaki et al. 2013, Yoshino et al. 2013). Our results clearly demonstrate the structural similarity in glycosylation of the sporocysts of *F. hepatica* and the surrounding hepatopancreas, genital glands and renopericardial tissues, including mannose/glucose and N-acetylglucosamine residues bound by LCA, WGA and LEL.

The rediae of *F. hepatica* move freely between snail tissues. At the redial surface we identified mannose/glucose, N-acetylglucosamine and N-acetylgalactosamine residues. Obviously, the lectin binding pattern of the sporocysts and rediae of *F. hepatica* is different as of rediae there is a lack the sites for ConA and PNA binding. At the same time, there appear sites for PNA on the tubular epithelium of the hepatopancreas tissue. The observed change of surface glycosylation of the different larval stages in the snail body probably provides different types of interactions with host defense molecules and cells determining immune evasion. However, studies with lectin binding and monoclonal antibodies to carbohydrate epitopes in *Himasthla elongata*-*Littorina littorea* (Iakovleva & Gorbushin 2005) and *Schistosoma mansoni*-*Biomphalaria glabrata* (Zelck & Becker 1990 , Lehr et al. 2008) showed the presence of structural similarity of surface carbohydrates between redial/daughter sporocystic stages and surrounding host tissues. It was suggested that carbohydrate mimicry is one of the main mechanisms preventing adhesion of effector cells to the tegument of rediae/daughter sporocysts in these parasite-host associations.

We observed the abundance of sites for LCA, WGA and LEL on protruding parts (head collar and lateral projections) of the redial body. It is possible, due to the closer contact with snail tissues that these areas are further “masked” with carbohydrates, as suggested by Van Remoortere et al. (2000).

The cercariae of *F. hepatica* differ essentially from the sporocysts and the rediae in binding of particular lectins. On the cercarial body surface we found the presence of non-reducing residues of mannose and/or glucose, N-acetylglucosamine, N-acetylgalactosamine and galactose, recognised respectively by ConA, WGA, SBA and PNA. Furthermore, the glycosylation of the body and tail surface is different, as found in other studied trematode species (Nanduri et al. 1991, Horák 1995, Horák & Mikeš 1995, Iakovleva & Gorbushin 2005 , Podhorský et al. 2009). Cercariae of *F. hepatica* spend a short time in the intermediate host. Under appropriate conditions, the mature cercariae leave the rediae across the birth opening and migrate via the snail circulatory system to the peripheral sinuses of the mantle (Ginetsinskaya 1968 , Andrews 1999). Here, despite the demonstrated similarity in labelling with WGA and PNA on snail tissues and the cercarial surface, as a short-living stage inside the snail the cercariae probably rely less on carbohydrate similarity for protection, but rely more on their mobility and on, perhaps, other mechanisms for immune evasion, as suggested by Iakovleva & Gorbushin (2005).

In our study we did not find fucose residues on the surface of sporocysts, rediae and cercariae of *F. hepatica*. This monosaccharide residue was not found on *F. hepatica* miracidia either (Georgieva et al. 2012). At the same time, fucose is demonstrated in all studied tissues of *G. truncatula*. The presence of fucose-containing carbohydrate structures on the snail host might explain the different molluscan hosts of *Schistosoma mansoni* and *S. japonicum*, as suggested by Lehr et al. (2010).

In conclusion, the data presented here clearly demonstrate an interaction of lectins with snail tissues and the surface of sporocysts, rediae and cercariae of *F. hepatica*. This provides evidence for the structural similarity of carbohydrate residues in the contact zone between both organisms, suggesting that carbohydrate mimicry is utilised by the parasite as an evasion strategy in *G. truncatula - F. hepatica* system.
